# 2292: Implementation and dissemination of a unique training program in stem cell biology and regenerative medicine

**DOI:** 10.1017/cts.2017.170

**Published:** 2018-05-10

**Authors:** Matthew Jones, David Felson, David Center, Darrell Kotton

**Affiliations:** Boston University, Boston, MA, USA

## Abstract

OBJECTIVES/SPECIFIC AIMS: Provide an innovative, integrative, and interdisciplinary training program which will leverage a unique and internationally recognized strength of BU and establish an environment that facilitates translational team science interactions with MD scientists and clinicians, thereby synergistically bridging research strengths with interdisciplinary approaches. METHODS/STUDY POPULATION: This overall mission of the RMTP is pursued through 2 independent aims. Aim 1: Provide an innovative, integrative, and interdisciplinary training program which will leverage a unique and internationally recognized strength of BU. Aim 2: Establish an environment that facilitates translational team science interactions with MD scientists and clinicians, thereby synergistically bridging research strengths with interdisciplinary approaches. To achieve these aims, we have developed a specialized didactic curriculum that is fully integrated in graduate school training and can be shared for the benefit of others outside of the BU community. We are also developing online iPSC practicum workshops for more efficient distribution of didactic content. Interdisciplinary team science approaches to stem cell research related to cures for human diseases are fostered across investigators across diverse hubs at BU, BU Medical Center, the Charles River Campus and the Framingham Heart Study. All methodology, data and materials are provided in a transparent and open-source manner to benefit the greater scientific community and ensure rigorous reproducibility. RESULTS/ANTICIPATED RESULTS: As a nascent TL1 training program, we are just arriving at the end of our second year. At this point, 5 out of a total of 11 appointed trainees have concluded RMTP support, all of whom have transitioned into biomedical science-related pursuits; 2 predoctoral trainees were awarded F31 fellowships, 2 postdoctoral trainees were awarded career transition grants (K99/R00 and LERN fellowship), and 1 postdoctoral trainee became a Senior Scientist at a Biopharmaceutical company. Given the quality of our trainees and their RMTP mentors, we anticipate that close to 100% of those supported by this mechanism will continue their career development in the biomedical sciences. DISCUSSION/SIGNIFICANCE OF IMPACT: Implementation of the RMTP TL1 would not only serve to increase the capacity of trainees within the CReM, but would also extend the scope of regenerative medicine research to other CTSI-participating hubs and more broadly to other scientific disciplines.

